# An Anomalous Case of Midwifery

**Published:** 1889-09

**Authors:** D. B. McGee

**Affiliations:** May, Texas


For Daniel's Texas Medical Journal.

### AN ANOMALOUS CASE OF MIDWIFERY.

BY D. B. M'GEE, M. D., MAY, TEXAS.

IF YOU WILL kindly lend me space in your valuable journal, I will be pleased to report a unique case of obstetrics that occurred to me very recently, with the hope of gaining some information, more than anything else.

CASE.—Mrs. D———, aet 18 years, primipara, brunette, large, physique, previous health and family history good. Was called to attend her in her first confinement, July 5, current year, liquor amnii having escaped before I arrived. Immediately made examination which revealed vertex presentation all right, but on palpation of uterine tumor found same exceedingly large (notwithstanding previous escape of liquor amnii) and very tympanitic; uterine contractions rather feeble; gave quiniæ sulphat. grs, xv, which increased force and frequency. I again made vaginal examination during acme of a pain, and instructed my patient to "bear down," when, to my great surprise and wonder, a considerable amount of gas escaped per vagina. This set me to thinking! But I could in no way account for it unless there be a fistulous tract communicating with the uterus and "some cavity." All efforts to find such a fistulous tract proved futile. Will add that after completion of second stage, another gaseous expulsion ensued, eliciting a sound not unlike that of flatus escaping from the rectum. When placenta came away, there was also gas imprisoned between it and membranes.

The labor, was, with this strange exception, a perfectly natural one, the lady being in due time delivered of a perfectly healthy, well developed male child, without an untoward symptom, and both are doing well.

I hope that some learned brother may enlighten me, through the JOURNAL, as to how gas could enter a gravid uterus without being hurtful to fœtus, and indeed without a thoroughfare.

